# Adaptive radiation and structural tailoring of the Vietnamese *Blec2* immunogenetic reservoir

**DOI:** 10.3389/fgene.2026.1819401

**Published:** 2026-05-07

**Authors:** Anh Huynh Luu, Trifan Budi, Worapong Singchat, Chien Tran Phuoc Nguyen, Thitipong Panthum, Nivit Tanglertpaibul, Kanithaporn Vangnai, Aingorn Chaiyes, Chotika Yokthongwattana, Chomdao Sinthuvanich, Orathai Sawatdichaikul, Kyudong Han, Narongrit Muangmai, Darren K. Griffin, Prateep Duengkae, Ngu Trong Nguyen, Kornsorn Srikulnath

**Affiliations:** 1 Animal Genomics and Bioresource Research Unit (AGB Research Unit), Faculty of Science, Kasetsart University, Bangkok, Thailand; 2 Interdisciplinary Graduate Program in Bioscience, Faculty of Science, Kasetsart University, Bangkok, Thailand; 3 College of Agriculture, Can Tho University, Can Tho, Vietnam; 4 School of Agricultural Technology, King Mongkut’s Institute of Technology Ladkrabang, Bangkok, Thailand; 5 Department of Forest Biology, Special Research Unit for Wildlife Genomics (SRUWG), Faculty of Forestry, Kasetsart University, Bangkok, Thailand; 6 Department of Food Science and Technology, Faculty of Agro-Industry, Kasetsart University, Bangkok, Thailand; 7 The International Undergraduate Program in Bioscience and Technology, Faculty of Science, Kasetsart University, Bangkok, Thailand; 8 Department of Biochemistry, Faculty of Science, Kasetsart University, Bangkok, Thailand; 9 Department of Nutrition and Health, Institute of Food Research and Product Development, Kasetsart University, Bangkok, Thailand; 10 Department of Microbiology, College of Bio-convergence, Dankook University, Chungnam, Republic of Korea; 11 Bio-Medical Engineering Core Facility Research Center, Dankook University, Chungnam, Republic of Korea; 12 Smart Animal Bio Institute, Dankook University, Chungnam, Republic of Korea; 13 Department of Fishery Biology, Faculty of Fisheries, Kasetsart University, Bangkok, Thailand; 14 School of Biosciences, University of Kent, Canterbury, United Kingdom; 15 Biodiversity Center Kasetsart University (BDCKU), Bangkok, Thailand

**Keywords:** AlphaFold 3, HPAI H5N1 resistance, Marker-Assisted Selection, *MHC-B* locus, red queen hypothesis

## Abstract

**Background:**

The global poultry industry faces a critical sustainability crisis driven by climate change and escalating disease threats, necessitating the identification of novel genetic reservoirs for resilience. The Major Histocompatibility Complex (*MHC*)-linked *Blec2* gene, a member of the Killer Cell Lectin-like Receptor (KLR) family, serves as a molecular sentinel regulating Natural Killer (NK) cell-mediated immunity; however, its diversity remains poorly characterized in Southeast Asian avian populations.

**Methods:**

This study utilized Illumina short-read sequencing and AlphaFold 3 modeling to investigate *Blec2* polymorphism across 15 Vietnamese populations that comprised 13 indigenous breeds and two ancestral Red Junglefowl (*Gallus gallus*) lineages.

**Results:**

We identified 11 unique alleles, including the novel variant *VN11*, that establish the Vietnamese gene pool as a distinct reservoir of immune diversity. Notably, eight alleles were restricted to indigenous breeds, which exhibited a higher nucleotide diversity (*π* = 0.012) than their wild progenitors (*π* = 0.009), suggesting that localized diversification has been driven by breed-specific selective pressures. Evolutionary analysis revealed a dual mechanism: (1) intense global purifying selection (*ω* ≤ 0.035) preserving the receptor’s structural scaffold and (2) localized positive selection at codons 11 and 18 that is predicted *in silico* to influence the receptor’s binding affinity for the *MHC* Class I (BF2) ligand. The hypothesized functional relevance of this diversity is highlighted by the identification of alleles homologous to haplotypes previously associated with high resistance, such as Blec2*VN2 (linked to H5N1 and Marek’s disease resistance in prior studies) and Blec2*VN9 (associated with respiratory virus resilience).

**Conclusion:**

By integrating population genomics with *in silico* structural biology, this study provides the foundational data proposing *Blec2* as a candidate locus for future Marker-Assisted Selection (MAS) research. While experimental functional validation remains necessary, our findings establish a hypothesis-driven framework for investigating how genetic reservoirs might be leveraged to enhance avian immunocompetence and secure sustainable poultry production against emerging viral threats.

## 1 Introduction

The poultry industry, which serves as a vital global food source characterized by rapid annual growth exceeding 4% (Govoni et al., 2021) and encompasses nearly 1,600 recognized breeds (Ekarius, 2007; Knowles et al., 2008; Bello et al., 2023), is currently facing a critical sustainability crisis driven by climate change and escalating global temperatures (Mirón et al., 2023). Because poultry lack sweat glands, they are highly susceptible to heat stress, the physiological impacts of which severely impair immune response, growth, and reproductive efficiency (Tamzil, 2014; El-Kholy et al., 2017; Aryani et al., 2019; Alagawany et al., 2017; Kumar et al., 2021). This environmental pressure is compounded by increased disease prevalence and resource scarcity, which further compromise the industry’s efficiency and lead to elevated mortality rates (Soliman and Safwat, 2020; Godde et al., 2021; Attia et al., 2024; Ngongolo and Mrimi, 2024). While modern intensive selection has prioritized production traits, it has inadvertently eroded the ancestral genetic variation originally derived from the red junglefowl (RJF), necessitating an urgent focus on the conservation and characterization of local chicken gene pools (Chebo et al., 2022). Regional initiatives, such as the Siam Chicken Bioresource Project, have highlighted Southeast Asian indigenous chickens, including phenotypically distinct Vietnamese breeds like the Dong Tao, Hmong, and Ac, as critical reservoirs of genetic diversity that have been shaped by centuries of rigorous natural and local selection. These populations, which represent an essential ancestral baseline, likely harbor unique alleles associated with resilience against endemic pathogens and environmental stressors. Despite the mounting pressure of transboundary viral threats, such as highly pathogenic avian influenza (HPAI) and Marek’s disease (MD), the specific immunogenetic regions responsible for this observed resilience in Vietnamese breeds remain largely uncharacterized. Therefore, making use of advanced genomic technologies to identify beneficial resistance traits within these conserved gene pools (Van et al., 2020; Olaniyan et al., 2024) is a fundamental strategy for fortifying the avian defense system and securing poultry production against future global challenges.

The field of avian immunogenetics is fundamental to sustainable poultry production as it leverages the genetic basis of disease resistance encoded by a complex network of immune-related genes (Burgess, 2004; Almajwal et al., 2018; Nonić, 2020). Central to this system is the Major Histocompatibility Complex (*MHC*), an ancient and compact gene cluster on a microchromosome that exhibits extreme genetic variability to distinguish self from non-self-entities (Miller et al., 1996; Nikbakht et al., 2022). In chickens, the *MHC* is identified as the B blood group system, which consists of the *MHC-B* and *MHC-Y* regions (Briles and Briles, 1987; Kaufman et al., 1999). The *MHC-B* core is particularly critical, as it harbors essential genes such as *BLB1*, *BLB2*, *BF1*, and *BF2*, alongside the putative Natural Killer (NK) cell receptor, *Blec2* (also known as B-NK) (Kaufman, 1999; Shiina et al., 2007; Ediriweera et al., 2022). Polymorphisms within this region directly determine host susceptibility to devastating pathogens, including MD and Infectious Bursal Disease Virus (IBDV), while simultaneously correlating with economic traits such as growth and egg production (Nikbakht and Esmailnejad, 2015; Haunshi et al., 2020; Mehrzad and Houshmand, 2025). As a member of the Killer Cell Lectin-like Receptor (KLR) family, the *Blec2* gene functions as a molecular sentinel on NK cells and T-cell subsets, where it regulates both innate and adaptive immunity (Viertlboeck et al., 2008; Hao et al., 2021). Recent evidence underscores the clinical importance of this locus, specifically identifying certain *Blec2* haplotypes that are probably associated with resistance to HPAI H5N1, whereas others are linked to high mortality (Budi et al., 2025). This intense selection pressure is reflected in the high frequency of non-synonymous single-nucleotide polymorphisms (SNPs) within the gene’s ligand-binding domain. Given that indigenous chicken breeds serve as essential genetic resources by retaining natural disease resistance (Bettridge et al., 2018), the fine-scale characterization of *Blec2* polymorphism in the Vietnamese indigenous reservoir and its wild ancestor, the RJF, is paramount (Rogers and Kaufman, 2008). Integrating *MHC*-based Marker-Assisted Selection (MAS) into breeding strategies is therefore an essential step to enhance poultry immunocompetence against evolving pathogen threats and to preserve the diversity discovered within these local populations (Mehrzad and Houshmand, 2025).

Indigenous chickens typically exhibit greater genetic diversity than modern commercial breeds, as they have not been subjected to the intensive artificial selection that has led to a significant erosion of genetic diversity in industrial stocks (Araujo de Carvalho et al., 2020; Chebo et al., 2022; Costamilan et al., 2025). In this context, Vietnamese indigenous and local breeds and their progenitor, the RJF, constitute a unique reservoir of natural genetic variability that is essential for securing global poultry production (Cuc et al., 2025). These resilient populations, which account for 60%–65% of Vietnam’s national chicken flock (Lan Phuong et al., 2015), possess crucial survival traits, including superior adaptability to harsh environments and high disease resistance (Buranawit and Laenoi, 2021; Nguyen et al., 2022). This high genomic variability extends prominently to immune system loci; for instance, studies on Brazilian Caipira chickens identified 23 alleles within the *MHC* B-F genes, ten of which were entirely absent in commercial lines (Lima-Rosa et al., 2005). However, a significant research problem persists: Despite the known polymorphism of *Blec2* in neighboring regions, such as the 14 distinct alleles recently reported in Thai indigenous chickens (Budi et al., 2025), the fine-scale allelic diversity and evolutionary trajectory of this molecular sentinel within Vietnamese populations remain entirely unresolved. This knowledge gap creates a barrier to the development of effective MAS programs tailored to the unique pathogen landscape of Southeast Asia. To address this, the *Blec2* intron 3/exon 4 region was selected for its high polymorphism in Galliformes (Shiina et al., 2007; Hosomichi et al., 2008) and its structural importance. This region encodes the C-type lectin-like domain (Kelley and Trowsdale, 2005; Viertlboeck et al., 2008), which serves as the molecular interface for *MHC* Class I (*BF2*) recognition (Straub et al., 2013). Non-synonymous mutations within this domain alter the tertiary structure of the binding pocket, modulating receptor affinity and tuning *NK* cell-mediated response thresholds (Rogers and Kaufman, 2008; Zhang et al., 2024). By targeting this site of host-pathogen evolutionary arms races (Těšický and Vinkler, 2015; Lighten et al., 2017), the amplicon provides a framework for computationally evaluating adaptive diversity, though definitive phenotypic implications require future validation. Therefore, the central research question of this study asks: To what extent have localized selective pressures and ancestral lineage sorting shaped the unique *Blec2* allelic repertoire and protein conformations in Vietnamese indigenous chickens compared to their wild counterparts? We hypothesized that geographically isolated Vietnamese breeds possess a distinct *Blec2* diversity, the polymorphisms of which confer structural advantages for pathogen recognition. To test this, we analyzed 15 distinct populations (13 indigenous and two RJF), which served as our independent variables, while treating the resulting allelic frequencies, selection signatures, and predicted protein conformations as our dependent variables. By leveraging Illumina sequencing and AlphaFold 3 modeling, this study aims to bridge the current genomic gap, providing the foundational data necessary to enhance innate immune capabilities and secure sustainable poultry production against emerging disease threats.

## 2 Materials and methods

### 2.1 Sample acquisition and genomic DNA preparation

Ethical approval for all experimental protocols was granted by the Kasetsart University Animal Experiment Committee (Approval No. ACKU67-SCI-021), which adhered to Kasetsart University Animal Experiment Regulations and the ARRIVE guidelines (https://arriveguidelines.org/). Blood samples were obtained from 15 distinct chicken populations across Vietnam, comprising 13 indigenous/local populations and two populations of RJF, the details of which are documented in Supplementary Table 1; Supplementary Figure 1. Following the formal consent of farm owners, whole blood was drawn from the brachial vein under manual restraint using Vacuette® 21-gauge needles and transferred into tubes containing 5 mM EDTA (Greiner Bio-One, Kremsmünster, Austria). All sampled chickens, which were handled according to welfare standards, were immediately released into their original environments post-collection. The samples were maintained at 4 °C until genomic DNA was extracted via a standard salting-out procedure (Supikamolseni et al., 2015). To ensure suitability for downstream analysis, DNA quality was rigorously assessed using a NanoDrop 2000 spectrophotometer (Thermo Fisher Scientific, Wilmington, DE, USA) and 1% agarose gel electrophoresis, which verified both the concentration and structural integrity of the isolates.

### 2.2 Targeted enrichment of the *Blec2* locus and Salus Pro short-read sequencing library construction

A partial region of the *Blec2* gene, which encompasses intron 3 and exon 4, was targeted for analysis because this locus is known to exhibit significant polymorphism across the order Galliformes (Budi et al., 2025). Genomic DNA (approximately 25 ng) was amplified via polymerase chain reaction (PCR) using the primer pair pcBlec2F (5′-GAC​AGA​GCA​GGC​AGG​CAG​CA-3′) and pcBlec2R (5′-GGG​CTG​CAA​CCA​CCC​CAG​TT-3′), the sequences of which followed the established protocol of Eimes et al. (2013). To facilitate multiplexed identification during high-throughput sequencing, unique 8-base pair (bp) barcode sequences (Macrogen Inc., Seoul, Korea) were appended to the 5′end of each forward primer. Each 15 μL reaction volume contained 1× Apsalagen buffer (supplemented with 1.5 mM MgCl_2_), 0.2 mM dNTPs, 0.5 μM of each primer, and 0.5 U of Taq DNA polymerase (Apsalagen Co., Ltd., Bangkok, Thailand). The thermocycling profile consisted of an initial denaturation at 94 °C for 5 min, followed by 35 cycles of 94 °C for 30 s, 58 °C for 30 s, and 72 °C for 30 s, which concluded with a final 5-min extension at 72 °C. To ensure high fidelity and mitigate the risk of false allele detection, each sample was amplified in triplicate before amplicons were verified via 2% agarose gel electrophoresis. For the sequencing phase, 92 PCR products per barcode set were pooled into six distinct libraries, which were subsequently subjected to paired-end sequencing on a SeqStudio™ Genetic Analyzer (Thermo Fisher Scientific Inc., Waltham, MA, United States) at the Kasetsart–Salus Biomed–Gibthai Collaborative Excellence Center, Bangkok, Thailand.

### 2.3 Raw read curation and computational assignment of barcoded reads

The 196-bp paired-end reads were initially subjected to quality control using FASTQC version 0.12.0 (Andrews, 2010). This diagnostic step evaluated essential parameters to ensure adherence to stringent quality standards prior to the merging and demultiplexing of sequences from the pooled libraries, specifically confirming a high per-base sequence quality (Phred Q > 30) and the absence of adapter contamination. Subsequent allelic assignment for the *Blec2* gene was conducted via the AmpliSAS pipeline (Sebastian et al., 2016), which utilized several filtering parameters to ensure data reliability and mitigate sequencing artifacts. Chimeric sequences and PCR artifacts were filtered using the AmpliSAS version 1.0, which identifies putative chimeras that can be reconstructed from two higher-abundance parent sequences within the same sample. These filters included a minimum read depth threshold of 100 and a maximum limit of six alleles per individual, the latter of which reflects potential duplicated *Blec2* loci as previously characterized by He et al. (2022). Additionally, the Degree of Change (DOC) parameter was employed to differentiate authentic alleles from stochastic artifacts based on read depth distribution (Lighten et al., 2014), while all other AmpliSAS parameters were maintained at default settings. The resulting individual sequences were analyzed for homology through BLASTn searches against the National Center for Biotechnology Information (NCBI) database, which facilitated the precise identification of the *Blec2* locus. All sequences were subsequently aligned and translated into their corresponding amino acid sequences using Geneious Prime version 2024.0.5 (https://www.geneious.com/), which confirmed the absence of premature stop codons within the exon 4 coding region of all identified alleles. Finally, the sequence data were deposited in a public data repository, specifically the NCBI GenBank database, under the accession numbers PX994335–PX994345 (https://www.ncbi.nlm.nih.gov/, accepted on 12 February 2025).

### 2.4 Evolutionary reconstruction and phylogenetic inference of *Blec2* alleles

Phylogenetic relationships among the *Blec2* alleles were inferred using Bayesian Inference (BI) as implemented in MrBayes version 3.2.6 (Ronquist and Huelsenbeck, 2003). Prior to the analysis, the optimal nucleotide substitution model was determined via ModelFinder (Kalyaanamoorthy et al., 2017), which is integrated within the IQ-TREE software and identifies the most suitable model based on the lowest Bayesian Information Criterion (BIC) score. The Bayesian analysis employed a Markov Chain Monte Carlo (MCMC) algorithm with four independent chains running for one million generations, the trees from which were sampled every 100 generations once log-likelihood values achieved stationarity. This process yielded 10,000 trees that were used to construct a majority-rule consensus tree featuring mean branch lengths, after the initial samples from 10% burn-in (100,000 generations) phase were discarded. For global comparative context, partial *Blec2* gene sequences encompassing exon 4 and intron 3 from diverse chicken breeds were retrieved from a public data repository (NCBI) *via* BLASTn searches. The resulting phylogeny was visualized using the Interactive Tree of Life (iTOL) online version 5 (Letunic and Bork, 2021). To assess genetic differentiation among populations, a Principal Coordinate Analysis (PCoA) was performed based on allele frequency data, with pairwise genetic distances calculated using the poppr package in R software version 4.5.2 (R Core Team, 2025). Moreover, allelic relationships were elucidated using a median-joining network algorithm in PopART version 1.7 (http://popart.otago.ac.nz), which utilized aligned nucleotide sequences to represent alleles as nodes to facilitate the visualization of shared alleles and population-specific variants (Bandelt et al., 1999).

### 2.5 Quantification of genetic diversity and signatures of molecular selection

Genetic diversity was quantified by estimating the number of alleles (*N*_a_) and nucleotide diversity (*π*) using DnaSP version 6.12 (Rozas et al., 2017). The average rates of synonymous (*dS*) and nonsynonymous (*dN*) substitutions per site were calculated via the Nei–Gojobori method (Nei and Gojobori, 1986) with Jukes–Cantor correction, which was followed by a *Z*-test to determine the *dN*/*dS* ratio (*ω*). To account for multiple testing across population comparisons, raw p-values derived from the *Z*-tests were subjected to a Benjamini–Hochberg False Discovery Rate (FDR) correction. In this framework, *ω* values approaching 1 were interpreted as evidence of neutral evolution, while *ω* > 1 and *ω* < 1 indicated positive and purifying selection, respectively. Potential evolutionary constraints were further interrogated through neutrality tests, including Tajima’s *D*, Fu’s *F*, Fu and Li’s F*, and Fu and Li’s D*, which are based on allelic frequency spectra and were also conducted in DnaSP. To identify site-specific selection signals across the *Blec2* gene, codon alignments (comprising 11 sequences and 31 codons) were analyzed using the suite of models available on the Datamonkey web server (https://www.datamonkey.org/). Episodic positive selection was pinpointed using the Mixed Effects Model of Evolution (MEME), the sites of which were identified via a likelihood ratio test (LRT) with a significance threshold of *p* ≤ 0.01 (Murrell et al., 2012; Weaver et al., 2018). Pervasive selection was detected using two complementary methods: Fixed Effects Likelihood (FEL), which estimated *dN* and *dS* rates at each site across the phylogeny using a threshold of *p* ≤ 0.01 (Kosakovsky Pond and Frost, 2005), and Fast Unconstrained Bayesian Approximation (FUBAR). The latter, which is a computationally efficient Bayesian approach suited for detecting weak and widespread selection, inferred significance based on a posterior probability ≥0.9 (Murrell et al., 2013). This integrated bioinformatic approach enabled the robust detection of both intermittent and constant selective pressures acting upon the *Blec2* codon sites.

### 2.6 *In silico* structural modeling and conformational analysis of *Blec2* alleles

The tertiary (three-dimensional; 3D) protein structure of the *Blec2* exon 4 region was predicted using the AlphaFold 3 server version AF3 (https://alphafoldserver.com/), which utilizes a diffusion-based architecture to generate highly accurate molecular insights (Abramson et al., 2024). The resulting models were rendered using BIOVIA Discovery Studio version 24.1 (Dassault Systèmes, San Diego, CA, USA) and subjected to rigorous quality assessment via PROCHECK version 3.5.4 (Laskowski et al., 1993), which generated Ramachandran plots to verify the stereochemical stability of the polypeptide backbone. The 3D protein model was constructed using the protein sequence from the Korean Ogye breed (Accession WBF70159) as a reference template. This reference shares a sequence identity ranging from 83.9% to 100% with the Vietnamese chicken *Blec2* alleles, which were the primary focus of the structural analysis. These validated structures were subsequently superimposed and contrasted with the reference lectin-like natural killer cell surface protein, the sequence of which was retrieved from the UniProt database (A5HUL0) (https://www.uniprot.org/), to identify structural deviations associated with the identified allelic variants.

## 3 Results

### 3.1 Characterization of *Blec2* allelic repertoires in indigenous and local chickens and RJF

**FIGURE 1:**
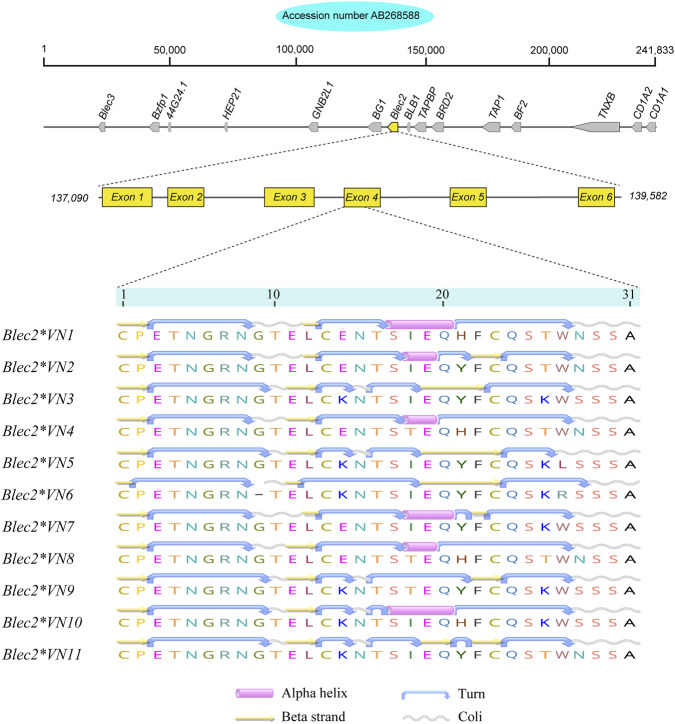
Secondary structures of *Blec2* protein variants predicted based on the amino acid sequences encoded by partial exon 4 fragments in Vietnamese indigenous/local chickens and red junglefowl.

**FIGURE 2:**
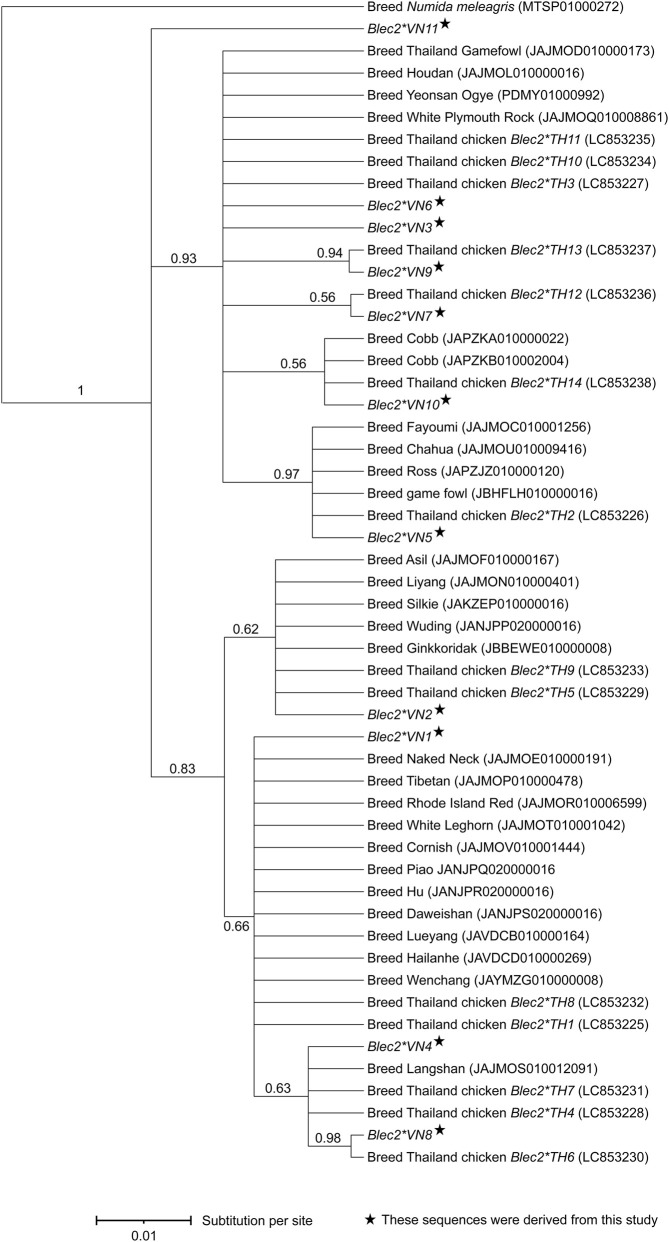
Bayesian phylogenetic tree of *Blec2* gene alleles for Vietnamese indigenous and local chicken breeds and red junglefowl. The values above the branches represent posterior probability. The scale shows substitutions per site.

**FIGURE 3:**
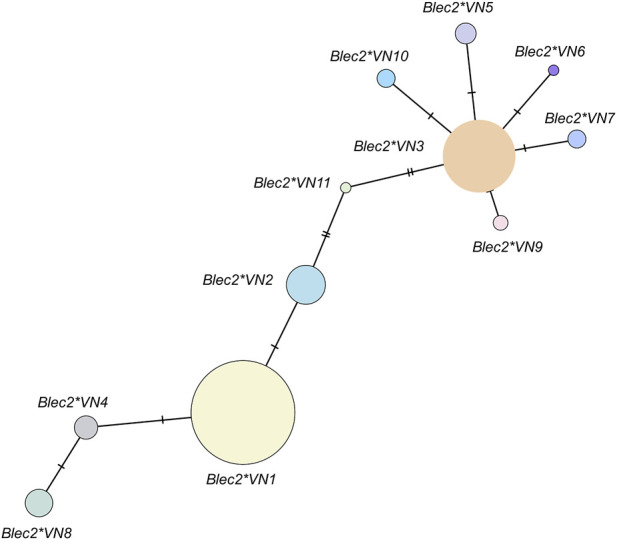
Allelic network depicting the relationships among *Blec2* gene variants found in indigenous Vietnamese chickens, local breeds, and red junglefowl. Each circle represents a unique allele, with its size proportional to allele frequency across the populations. Connecting lines indicate mutational steps, while short bars on these lines mark inferred mutational events. A specific color distinguishes each allelic node.

High-throughput short-read sequencing generated nucleotide sequences for a 196-bp fragment of the *Blec2* gene, which encompasses portions of intron 3 and exon 4 that encode characteristic alpha-helix-turn-beta-strand and coil motifs (Figure 1). Analysis of eleven variable sites revealed a total of 11 unique alleles, which included two novel variants (Blec2*VN6 and Blec2*VN11) and one allele (Blec2*VN1) that was identical to the established reference sequence (OM953777). While Blec2*VN1, Blec2*VN2, and Blec2*VN3 were the most prevalent alleles across both the Vietnamese indigenous/local chickens and the RJF, eight other alleles (Blec2*VN4 to Blec2*VN11) were restricted to the indigenous populations. Several of these displayed breed-specific distributions, such as Blec2*VN6 in the Ac chicken and Blec2*VN9 and Blec2*VN11 in the Hmong chicken (Supplementary Table S2). Sequence comparisons against global reference data identified eight mutational sites within the exon, which consisted of one synonymous (silent) and seven non-synonymous (missense) mutations, alongside two intronic sites characterized by a T>C transversion and a G>A transition (Table 1 and Supplementary Table S3). Genetic diversity was notably higher in the indigenous and local breeds, which exhibited a mean nucleotide diversity (*π*) of 0.012 and carried 2–8 alleles per population, whereas the RJF populations carried only 2–3 alleles with a lower mean *π* of 0.009 (Table 2). Bayesian phylogenetic reconstruction further indicated that the Blec2*VN11 allele occupies a distinct evolutionary branch (Figure 2). This genetic structure was supported by PCoA, where the first two major axes (PCo1 and PCo2) explained 36.2% and 11.7% of the total variation, respectively (Supplementary Figure S2). Moreover, the median-joining haplotype network (Figure 3) confirmed that while the Blec2*VN1 to Blec2*VN3 variants form the core ancestral cluster, the remaining haplotypes occur at lower frequencies and represent more recent diversification.

**TABLE 1:** Polymorphic sites within the *Blec2* gene alleles identified in this study.

Allele	Accession number	Intron 3		Exon 4							
		Nucleotide position*									
		9,648	9,671	9,695	9,698	9,699	9,701	9,717	9,725	9,738	9,753
Reference sequence	OM953777	T	G	T	C	A	G	G	A	C	C
*Blec2* * *VN1*	PX994335	.	.	.	.	.	.	.	.	.	.
*Blec2* * *VN2*	PX994336	.	.	.	.	.	.	A	.	.	.
*Blec2* * *VN3*	PX994337	.	A	C	.	.	T	A	.	T	.
*Blec2* * *VN4*	PX994338	.	.	.	.	.	.	.	G	.	.
*Blec2* * *VN5*	PX994339	.	A	C	A	.	T	A	.	T	.
*Blec2* * *VN6*	PX994340	.	A	C	.	T	T	A	.	T	-
*Blec2* * *VN7*	PX994341	.	A	C	.	.	T	A	.	.	.
*Blec2* * *VN8*	PX994342	C	.	.	.	.	.	.	G	.	.
*Blec2* * *VN9*	PX994343	.	A	C	.	.	T	A	G	T	.
*Blec2* * *VN10*	PX994344	.	A	C	.	.	T	.	.	T	.
*Blec2* * *VN11*	PX994345	.	A	.	.	.	.	A	.	T	.

* Nucleotide position based on reference sequence (accession number OM953777).

**TABLE 2:** Nucleotide sequence variation in the *Blec2* gene.

Breed/Red junglefowl subspecies	Population	*N* ^a^	*N*_a_ ^b^	*Π* ^c^
Ac	Tra Vinh	20	4	0.012
	Tien Giang	20	8	0.014
	Long An	20	4	0.013
Noi	Dong Thap	15	7	0.011
	Vinh Long	15	6	0.011
	Ben Tre	7	4	0.008
Tre	Can Tho	5	3	0.013
	Tra Vinh	13	4	0.011
	An Giang1	8	3	0.010
	An Giang2	5	2	0.011
Hmong	Hung Yen	15	8	0.014
Dong Tao	Hung Yen	11	4	0.010
Tau Vang	Ca Mau	15	4	0.013
*G. gallus spadiceus*	Kien Giang	10	2	0.008
*G. gallus*	An Giang	3	3	0.013
Indigenous and local breeds	169	11	0.012	​
Red junglefowl	13	3	0.009	​
Overall	182	11	0.012	​

^a^ number of samples (*N*).

^b^ number of maximum alleles per populations (*N*_a_).

^c^ nucleotide diversity (*π*).

### 3.2 Molecular selection analysis and codon-specific evolutionary constraints

The *Z*-test for selection revealed that the *Blec2* gene is under remarkable evolutionary pressure across most indigenous Vietnamese chicken breeds and RJF populations (Table 3). Global *ω* values ranged from 0.012 to 0.035, which indicates that strong purifying selection has shaped the contemporary genetic architecture of these populations to maintain functional protein stability. Within the RJF populations specifically, *ω* values remained low, ranging from 0.013 to 0.018. Neutrality tests, which included Tajima’s *D*, Fu and Li’s D*, and Fu and Li’s *F*, revealed further variations across the studied populations (Table 4). Specifically, Tajima’s *D* values ranged from −0.077 to 1.867, while Fu and Li’s D* and Fu and Li’s F* values ranged from −0.543 to 1.425 and −0.074 to 1.470, respectively; however, none of these metrics reached statistical significance, indicating that the populations have not undergone recent extreme expansions or bottlenecks. Codon-level selection analysis, which was performed using MEME on 11 aligned sequences and evaluated 13 branches per partition, initially indicated a predominance of neutral selection across the *Blec2* gene (Supplementary Table S4). Similarly, FEL analysis reinforced this finding of overall neutrality (Supplementary Table S5). By contrast, the more sensitive FUBAR method consistently identified signatures of positive selection at codons 11 and 18, the significance of which was supported by a posterior probability >0.9 (Supplementary Table S6). Moreover, FUBAR pinpointed codon 13 as being under significant negative (purifying) selection, which highlights the localized evolutionary constraints acting on specific amino acid residues despite the overall neutral background of the gene.

**TABLE 3:** Rates of synonymous (*d*_S_) and nonsynonymous (*d*_N_) substitutions in the nucleotide sequences of the *Blec2* gene.

Breed/Red junglefowl subspecies	Population	*d*_N_	*d*_S_	*ω* (*d*_N_/*d*_S_)	*Z*-test		
					*Z*-score	*p*-value	*p*-value (FDR)
Ac	Tra Vinh	0.0003	0.015	0.020	0.037	0.970	0.994
	Tien Giang	0.0003	0.011	0.028	−0.204	0.838	0.994
	Long An	0.0002	0.010	0.020	0.052	0.959	0.994
Noi	Dong Thap	0.0001	0.008	0.012	−0.443	0.659	0.994
	Vinh Long	0.0001	0.004	0.024	−0.566	0.572	0.994
	Ben Tre	0.0001	0.004	0.027	0.195	0.846	0.994
Tre	Can Tho	0.0002	0.011	0.018	0.166	0.869	0.994
	Tra Vinh	0.0001	0.005	0.022	−0.223	0.824	0.994
	An Giang1	0.0001	0.007	0.014	0.169	0.866	0.994
	An Giang2	0.0001	0.006	0.017	0.117	0.907	0.994
Hmong	Hung Yen	0.0001	0.003	0.035	−0.572	0.568	0.994
Dong Tao	Hung Yen	0.0001	0.007	0.015	0.070	0.945	0.994
Tau Vang	Ca Mau	0.0002	0.009	0.022	0.007	0.994	0.994
*G. gallus spadiceus*	Kien Giang	0.0002	0.015	0.013	0.123	0.902	0.994
*G. gallus*	An Giang	0.0003	0.017	0.018	0.179	0.858	0.994
Indigenous and local breeds	​	0.0001	0.004	0.023	−0.165	0.870	0.994
Red junglefowl	​	0.0001	0.007	0.014	0.151	0.880	0.994
Overall	​	0.0001	0.005	0.022	−0.157	0.876	0.994

To control for the false discovery rate across multiple population comparisons, raw *p*-values were adjusted using the Benjamini–Hochberg method. Adjusted *p*-values are reported in the final column. FDR: false discovery rate.

**TABLE 4:** Neutrality test for the *Blec2* gene sequences.

Breed/Red junglefowl subspecies	Population	Tajima’s *D*	Fu and Li’s *F*	Fu and Li’s *D*
Ac	Tra Vinh	1.867^ns^	0.972^ns^	0.368^ns^
	Tien Giang	0.960^ns^	−0.074^ns^	−0.543^ns^
	Long An	1.507^ns^	0.347^ns^	−0.260^ns^
Noi	Dong Thap	0.181^ns^	0.632^ns^	0.697^ns^
	Vinh Long	0.486^ns^	1.213^ns^	1.278^ns^
	Ben Tre	−0.077^ns^	0.453^ns^	0.563^ns^
Tre	Can Tho	1.057^ns^	1.459^ns^	1.425^ns^
	Tra Vinh	0.972^ns^	1.342^ns^	1.232^ns^
	An Giang1	0.700^ns^	1.247^ns^	1.234^ns^
	An Giang2	0.504^ns^	1.286^ns^	1.368^ns^
Hmong	Hung Yen	1.108^ns^	1.470^ns^	1.327^ns^
Dong Tao	Hung Yen	−0.038^ns^	−0.022^ns^	−0.011^ns^
Tau Vang	Ca Mau	1.822^ns^	1.006^ns^	0.466^ns^
*G. gallus spadiceus*	Kien Giang	0.258^ns^	1.097^ns^	1.214^ns^
*G. gallus*	An Giang	1.057^ns^	1.459^ns^	1.425^ns^
Indigenous and local breeds	​	1.436^ns^	0.909^ns^	0.365^ns^
Red junglefowl	​	1.021^ns^	1.305^ns^	1.171^ns^
Overall	​	1.410^ns^	0.891^ns^	0.354^ns^

ns, not significant.

### 3.3 Sequence-structure relationships and conformational modeling of *Blec2* alleles

The analysis of the *Blec2* gene focused on the exon 4 nucleotide sequences, which correspond to positions 9,684–9,779 of the reference sequence (OM953777) and encode 31 amino acids spanning residues 67 to 97. The resulting *Blec2* protein sequences exhibited high proteomic homology to the *Gallus gallus* reference (WBF70159), with sequence identities ranging from 90.3% to 100% and query coverage between 80% and 100%. Further homology analysis confirmed that these sequences maintained 90.3%–100% identity when compared against various breeds, including Ghagus, White Leghorn, and Korean native chicken, the details of which are visualized in Supplementary Figure S3. At codon 11, the substitution of Histidine with Tyrosine results in the loss of chemical reactivity (enzymatic potential), which is replaced by enhanced structural binding strength. Similarly, the substitution of Glutamate with Lysine at codon 18 introduces a chemically active handle, which creates a *de novo* binding site that considerably alters the predicted 3D structure of the protein (Supplementary Figure S3). 3D tertiary structures were generated for the *Blec2* alleles across six distinct models (Supplementary Figure S4), the structural integrity of which was assessed via Ramachandran plot analysis. This quality evaluation revealed variations in stereochemical stability that were likely driven by the specific amino acid compositions of each allele. Among these, Model Set 3 demonstrated the highest structural quality, with 84.6% of its residues positioned in the most favored (core) regions, followed by Model 6 and Model Set 4, which achieved 80.8% and 76.9% core residues, respectively. The remaining models (1, 2, and 5) exhibited lower structural resolution, with core residue values ranging between 65.4% and 73.1%. Notably, all predicted protein sequences were successfully matched to the *Blec2* protein template (A5HUL0), confirming the structural conservation of the locus across the identified variants (Supplementary Figure S5).

## 4 Discussion

Our characterization of the *Blec2* locus reveals that Vietnamese chickens harbor a diverse allelic repertoire, including novel variants Blec2*VN6 and Blec2*VN11. This is shaped by a dual evolutionary mechanism: a) rigid purifying selection (*ω* ≤ 0.035) preserving structural integrity, and b) localized positive selection at codons 11 and 18 driving hypothetically adaptive ligand recognition. These unique structural variants underscore the potential value of indigenous reservoirs for exploring strategies to enhance global poultry resilience against pathogens like HPAI H5N1.

### 4.1 Adaptive radiation and immunogenetic diversification of the *Blec2* allelic repertoire

The analysis of the 196-bp *Blec2* fragment firmly establishes the Vietnamese gene pool as a critical and distinct reservoir of immunogenetic diversity, the breadth of which reflects an evolutionary history shaped by regional isolation (Cuc et al., 2025; Budi et al., 2025). The discovery of 11 unique alleles, including the novel variant Blec2*VN11, represents a significant expansion of the known allelic repertoire for this *MHC*-B locus, which has historically shown lower levels of novelty in standardized commercial studies (Shiina et al., 2007; Hosomichi et al., 2008). These findings align with the paradigm that immune genes are among the most rapidly evolving classes in the vertebrate genome, where remarkable levels of polymorphism are maintained through co-evolutionary “arms races” with environmental pathogens (Hillier et al., 2004; Bustamante et al., 2005; Těšický and Vinkler, 2015). Notably, the *Blec2* gene exhibited a polyphyletic pattern in Bayesian phylogenetic and an admixed distribution in the PCoA, where no clear genetic clustering by breed or geography was observed. This structure is typical of the highly adaptive *MHC* region and indicates extensive historical admixture between the wild RJF and local domestic populations, the processes of which have been documented in several Vietnamese avian genomic studies (Nguyen-Phuc et al., 2016). The scattering of individuals across the genetic space suggests that the adaptive value of advantageous immune alleles likely facilitates their rapid spread across populations, thereby bypassing traditional geographic boundaries (Eizaguirre et al., 2012). This evolutionary dynamic is visually confirmed by the median-joining haplotype network, which illustrates common ancestral alleles as central hubs from which numerous low-frequency haplotypes branch independently. Such a topology represents the ongoing generation and retention of novel, adaptive immune variants, the accumulation of which provides the raw material for host–pathogen co-evolution (Lighten et al., 2017).

Given that *Blec2* is a pivotal member of the KLR family, which regulates NK-cell-mediated innate immunity and is associated with resistance to devastating diseases such as MD, the preservation of these localized genetic resources is essential for global biosecurity (Kelley and Trowsdale, 2005; Viertlboeck et al., 2008). The shared prevalence of Blec2*VN1, Blec2*VN2, and Blec2*VN3 between domestic breeds and the wild RJF identifies these as ancestral alleles, which have likely been maintained by purifying selection to provide broad-spectrum adaptive advantages against pervasive pathogens (Mukherjee et al., 2009; Zhu et al., 2014). This evolutionary continuity is further supported by the phylogenetic clustering of RJF and indigenous sequences, a relationship that has been similarly documented in other C-type lectin-like receptors across the order Galliformes (Haunshi et al., 2019). By contrast, the eight alleles restricted to Vietnamese indigenous populations (Blec2*VN4 to Blec2*VN11) suggest a process of localized adaptive radiation. This pattern likely stems from traditional, low-input backyard farming systems, the relaxed productivity constraints of which allow for the accumulation of rare variants in “allele-preserving islands” (Granevitze et al., 2009). This structural diversity contrasts sharply with the homogenization observed in industrial stocks, where intensive artificial selection often erodes the standing genetic variation necessary for immune-driven adaptation (Chebo et al., 2022). Notably, the identification of seven missense mutations within exon 4 across these unique alleles highlights a high capacity for functional variation, which serves as the raw material for host-pathogen interactions (Lighten et al., 2017). Genetic diversity metrics reinforce this distinction, as the higher nucleotide diversity (*π* = 0.012) and expanded allelic range (two to eight alleles per individual) in Vietnamese chickens compared to the RJF (*π* = 0.009) are hypothesized to serve as indicators of population resilience against emerging infectious diseases (Van et al., 2020; Nikbakht et al., 2022).

### 4.2 Purifying selection and the structural integrity of the *Blec2* molecular scaffold

The overarching evolutionary pressure acting on the *Blec2* locus is characterized by intense purifying selection. While *Z*-test p-values did not reach statistical significance (FDR-adjusted *p* = 0.994), likely due to the limited statistical power inherent in analyzing a short 196-bp fragment, the consistently low *ω* values (0.012–0.035) and high amino acid sequence identity strongly suggest evolutionary constraint acting to protect the gene’s essential biological function (Zhu et al., 2014). This rigorous negative selection is reflected in the high amino acid sequence identity (90.3%–100%) observed across global breeds, the conservation of which supports the role of *Blec2* as a non-redundant immune surveillance receptor (Afrache et al., 2020). As *Blec2* functions as an inhibitory NK receptor that recognizes *MHC* Class I (BF2), substitutions at critical residues are predicted *in silico* to significantly alter the receptor’s binding affinity or specificity for its ligand, a process that directly impacts NK cell regulation and host disease resistance (Straub et al., 2013). This mechanism aligns with recent structural paradigms, which suggest that *MHC*-related proteins can achieve distinct functional states through minimal amino acid modifications despite maintaining high overall sequence conservation (Zhang et al., 2024). Our AlphaFold 3 3D structural analysis of the 11 identified alleles further elucidated this relationship, as eight missense mutations resulted in six distinct protein models that suggest how subtle substitutions can reshape the receptor landscape. The stereochemical stability of these models, which was verified by high core residue values in Ramachandran plots (e.g., 84.6% in Model Set 3), supports the hypothesis that Vietnamese chicken populations maintain immune adaptability through precise conformational changes at the BF2-binding interface (Straub et al., 2013; Zhang et al., 2024). Consequently, while the primary lectin-like scaffold is preserved by evolutionary constraint to ensure functional folding, the identified *Blec2* variants represent promising candidates for future MAS research. Pending functional validation, such markers could potentially be explored to enhance innate disease resistance traits by optimizing the inhibitory signals that govern avian immune competence.

### 4.3 Positive selection signatures at codons 11 and 18: signatures of adaptive host–pathogen co-evolution in vietnamese chicken reservoirs

While global analysis indicated a background of purifying selection, codon-level interrogation *via* FUBAR revealed a more nuanced evolutionary landscape, identifying distinct signatures of positive selection at codons 11 and 18 and negative selection at codon 13. This structural polarization exemplifies the Red Queen Hypothesis (Lighten et al., 2017; Ye et al., 2023), where the gene’s core framework is conserved to maintain folding integrity, while specific residues at the host–pathogen interface evolve rapidly to sustain immune competence. The missense mutations identified at codons 11 and 18 are hypothesized to represent the “front lines” of this molecular conflict, the substitutions of which are computationally predicted to alter the protein’s tertiary stability and ligand-binding specificity (Rogers and Kaufman, 2008). Specifically, variants at codon 11 were documented in the Blec2*VN2, VN3, VN5, VN7, VN8, VN10, and *VN11* alleles, whereas codon 18 variants were restricted to the Blec2*VN2, VN3, VN8, VN9, VN10, and *VN11* lineages. The hypothesized functional significance of these variants is underscored by their homology with well-characterized *MHC* haplotypes that possess divergent disease-resistance profiles. Notably, Blec2*VN5 corresponds to the B12 haplotype, a genotype traditionally associated with increased susceptibility to bacterial lameness, Marek’s disease virus (MDV)-induced tumors, and specific strains of infectious bronchitis virus (IBV) (Plachy et al., 1992; Joiner et al., 2007; Banat et al., 2013). By contrast, Blec2*VN2 exhibits homology to the B21 haplotype, which is widely recognized as a primary determinant of genetic resistance. B21 homozygous individuals exhibit considerably lower rates of MDV-induced lymphoma and superior survival against H5N1 highly pathogenic avian influenza (Hansen et al., 1967; Boonyanuwat et al., 2006; Hunt et al., 2010; Bertzbach et al., 2022). Moreover, the Blec2*VN9 allele corresponds to the B5 haplotype (Supplementary Figure S6), which has been linked to the mitigation of clinical symptoms in infectious laryngotracheitis virus and demonstrates relative resistance to IBV Gray and M41 strains (Banat et al., 2013; Dunn et al., 2020). Although sequence homology suggests shared immunological advantages, functional equivalence remains theoretical. Future *in vivo* studies are required to determine if these structural similarities confer identical disease resistance in Vietnamese chicken alleles. Collectively, these findings highlight the potential of investigating these indigenous-derived alleles as candidate targets for future MAS programs, which, following experimental validation, may facilitate the development of resilient poultry lines capable of withstanding economically devastating infectious challenges.

### 4.4 Comparative immunogenomics: regional divergence and haplotype sharing between Vietnamese and Thai chicken reservoirs

The comparative immunogenomic landscape of the *Blec2* gene reveals a sophisticated evolutionary divergence between Vietnamese and Thai chicken populations, which function as indispensable *MHC-B* reservoirs that utilize distinct strategies to navigate intense pathogen-driven pressures. In Vietnam, domestic chickens exhibit a nucleotide diversity (*π* = 0.012) that significantly surpasses that of the RJF (*π* = 0.009), a disparity which supports a model of relaxed purifying selection and founder effects within isolated, low-input backyard systems that act as a “foundry” for allelic novelty, such as the unique Blec2*VN6 and Blec2*VN11 (Granevitze et al., 2009; Budi et al., 2023). This contrasts with the Thai “reservoir” model, where high, comparable diversity across domestic and wild cohorts (*π* approximately 0.018) is maintained by long-term balancing selection, which is statistically validated by a significant Tajima’s D value (2.337) and the retention of deep ancestral lineages like Blec2*TH8 and Blec2*TH10 (Hess and Edward, 2002; Nguyen-Phuc et al., 2016). While Z-tests confirm that purifying selection (*ω* < 1) remains the fundamental force preserving the gene’s essential scaffold, an observation reinforced by the absolute absence of synonymous mutations (*dS* = 0) in Thai RJF, the detection of positive selection at codons 11 and 18 via FUBAR analysis identifies these specific residues as active battlegrounds in a Red Queen conflict. Such site-specific diversification aligns with the mechanism of fluctuating selection, where spatially distinct pathogen landscapes, such as the endemic viral strains found in the Vietnamese tropics, drive genetic divergence that is unnecessary in non-endemic areas (Spurgin and Richardson, 2010; Manczinger et al., 2019). The functional consequences of this variation are further elucidated by 3D structural modeling, which predicts that Vietnamese alleles maintain high core stability (Model Set 3, 84.6% core residues) for precise receptor tuning, whereas Thai alleles associated with H5N1 resistance, such as Blec2*TH2, adopt partial loop-coil conformations. This adaptive structural instability is hypothesized to confer an evolutionary trade-off, where increased flexibility allows the NK cell receptor to explore alternative binding specificities for its *MHC* Class I ligand amidst rapidly mutating pathogens (Rogers and Kaufman, 2008; Straub et al., 2013). Moreover, the high mutational activity observed within exon 4 underscores its status as a functional hotspot, where the unique retention of RJF-specific lineages in Thailand versus the rapid, localized diversification in Vietnam highlights the high degree of geographic isolation and genetic specificity between these regions (Lighten et al., 2017; Budi et al., 2025). These findings suggest that the *Blec2* locus, situated within the critical *MHC-B* region, is a candidate determinant of disease susceptibility, where the Vietnamese gene pool provides highly specialized variants and the Thai reservoir offers a broad genetic repertoire. Such extensive polymorphism, which is frequently lost in commercial lines due to intensive artificial selection, underscores the vital importance of indigenous breeds as genetic insurance for the global poultry industry and the potential benefits of incorporating these unique immune profiles into future MAS programs (Manjula et al., 2021; Arango et al., 2024).

### 4.5 Navigating constraints and future directions for global poultry biosecurity

Despite the high-resolution genomic data provided by this study, certain limitations remain, the addressing of which will be critical for future validation. Specifically, while our focus on the intron 3 and exon 4 regions provided essential insights into the ligand-binding domain, future investigations should employ full-length gene sequencing to capture the complete structural landscape of the *Blec2* protein. Additionally, while AlphaFold 3 provides state-of-the-art structural predictions, *in vitro* functional assays, such as surface plasmon resonance or cell-based binding studies, are necessary to conclusively confirm how the identified variants interact with the *MHC* Class I (*BF2*) ligand. Moreover, although our 15-population sample captured remarkable regional diversity, expanding this dataset to include broader geographic reaches of Southeast Asia would further elucidate the migratory patterns and trans-border gene flow of ancestral *Blec2* alleles. A further limitation of this study is the restricted sample size for specific populations, which notably includes wild red junglefowl (*Gallus gallus*, *N* = 3) and certain local domestic cohorts (*N* = 5). While these numbers are statistically insufficient for definitive population genetic inferences, which typically require larger cohorts, these data provide a critical exploratory baseline for identifying allelic presence in these rare lineages. Validating the absolute allelic distributions will require significantly expanded future sampling efforts. By integrating high-throughput sequencing with *in silico* structural modeling, this study has bridged a critical gap in our understanding of the Vietnamese immunogenetic landscape, providing the foundational data to support the transition from broad-scale conservation to precision breeding. Our findings support the hypothesis that indigenous chickens have retained specific adaptive variants, such as the novel alleles found in Hmong and Ac chickens, that were likely lost in industrial stocks due to intensive artificial selection. By identifying alleles homologous to high-resistance haplotypes such as B21 and B5, this study offers a framework for a proactive, genetically based solution to mitigate the dual threats of climate change and emerging viral pathogens like HPAI H5N1. Subject to necessary functional validation, utilizing these “resilience alleles” as candidates within future MAS programs offers a potential sustainable strategy to fortify the global avian defense system, thereby ensuring the long-term food security of the poultry industry in the 21st century.

## 5 Conclusion

This study successfully addressed the critical research gap pertaining to the *Blec2* immunogenetic landscape in Vietnam by characterizing the allelic diversity and evolutionary trajectories of 15 distinct chicken populations. Our results provide a definitive answer to the central research question, revealing that localized selective pressures and ancestral lineage sorting have shaped a unique *Blec2* repertoire in Vietnamese indigenous chickens, the genetic breadth of which considerably exceeds that of their wild counterparts. We confirmed our hypothesis that these geographically isolated breeds possess distinct polymorphisms, specifically the novel variants Blec2*VN6 and Blec2*VN11, the structural configurations of which, as elucidated by AlphaFold 3 models, suggest specialized adaptations within the ligand-binding domain. By systematically evaluating the 15 populations, we demonstrated that the immunogenetic landscape, comprising the presence of unique alleles, selection signatures, and predicted protein conformations, is governed by a dual evolutionary mechanism. While we note that the exact allelic presence of our smallest cohorts remains exploratory due to sampling constraints, the overall dataset establishes a clear framework of rigid purifying selection (*ω* ≤ 0.035) that maintains the receptor’s structural integrity and localized positive selection at codons 11 and 18 that is predicted to drive adaptive recognition. The identification of alleles homologous to high-resistance B21 and B5 haplotypes further supports the hypothesis that Vietnamese indigenous chickens serve as a valuable genetic reservoir for potential disease resilience traits, many of which have been eroded in commercial stocks due to intensive artificial selection. This integration of high-throughput sequencing and *in silico* modeling bridges the current genomic gap, providing the foundational genomic and structural data necessary to guide future MAS research. These findings offer a hypothesis-driven pathway for potentially enhancing innate immunocompetence and securing sustainable poultry production against the intensifying global threats of emerging pathogens and climate change.

## Data Availability

The original contributions presented in the study are publicly available. All data generated or analyzed during this study are included in this published article and its supplementary information files. The sequences have been deposited in the NCBI GenBank database (https://www.ncbi.nlm.nih.gov/) under accession numbers PX994335 – PX994345.
