# Supplementary material for: Adaptive radiation and structural tailoring of the Vietnamese Blec2 immunogenetic reservoir
Source: Front Genet. 2026 May 7;17:1819401. doi: 10.3389/fgene.2026.1819401 (PMC13189733; doi:10.3389/fgene.2026.1819401)
Supplement: Supplementary file 1 [file DataSheet1.pdf]

Supplementary Material

1 Supplementary Figures and Tables

1.1 Supplementary Figures

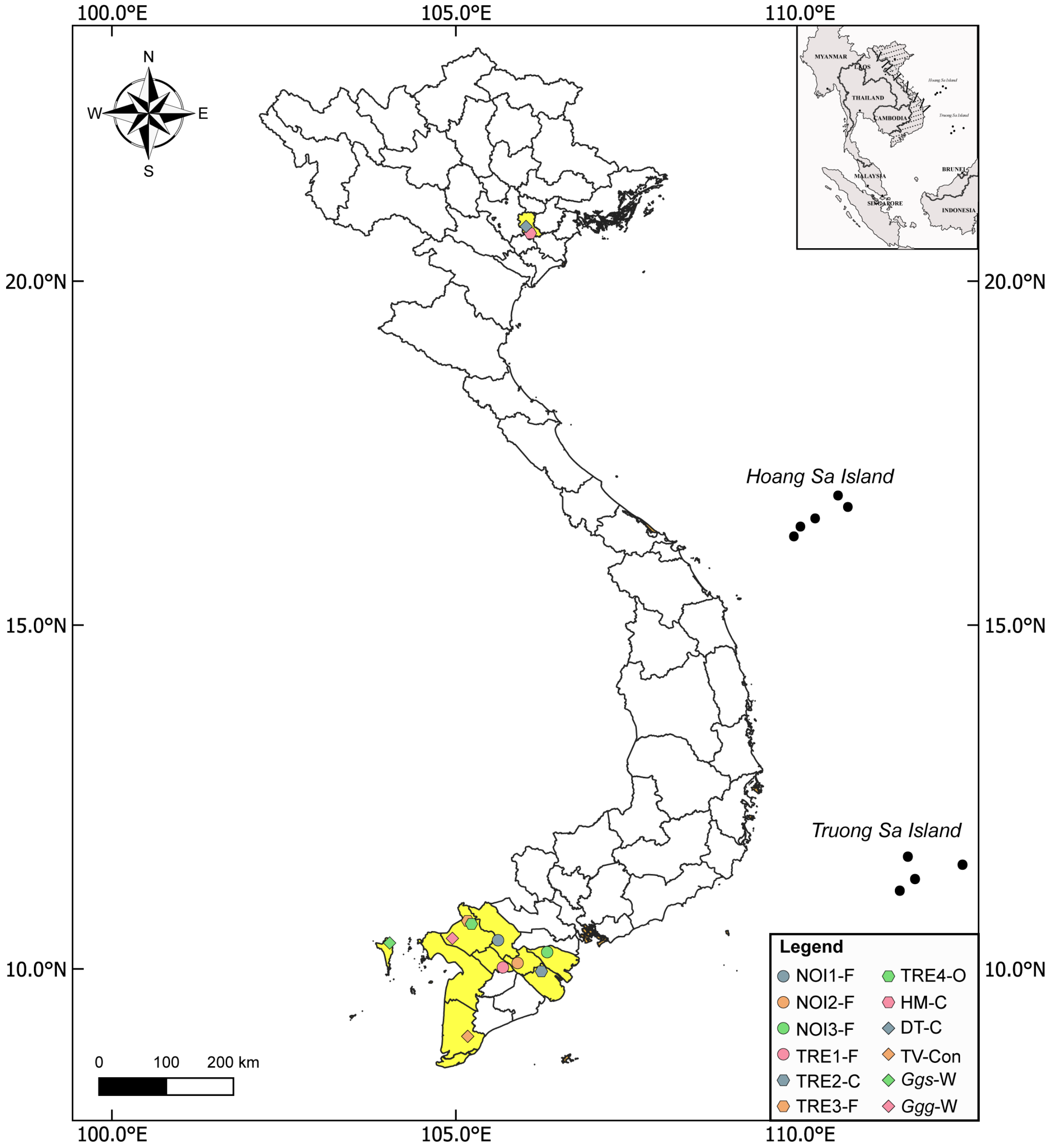

Supplementary Figure 1. Specimen details for 12 Vietnamese chicken populations, comprising the red jungle fowl and five native breeds (Noi, Tre, Hmong, Dong Tao, and Tau Vang)

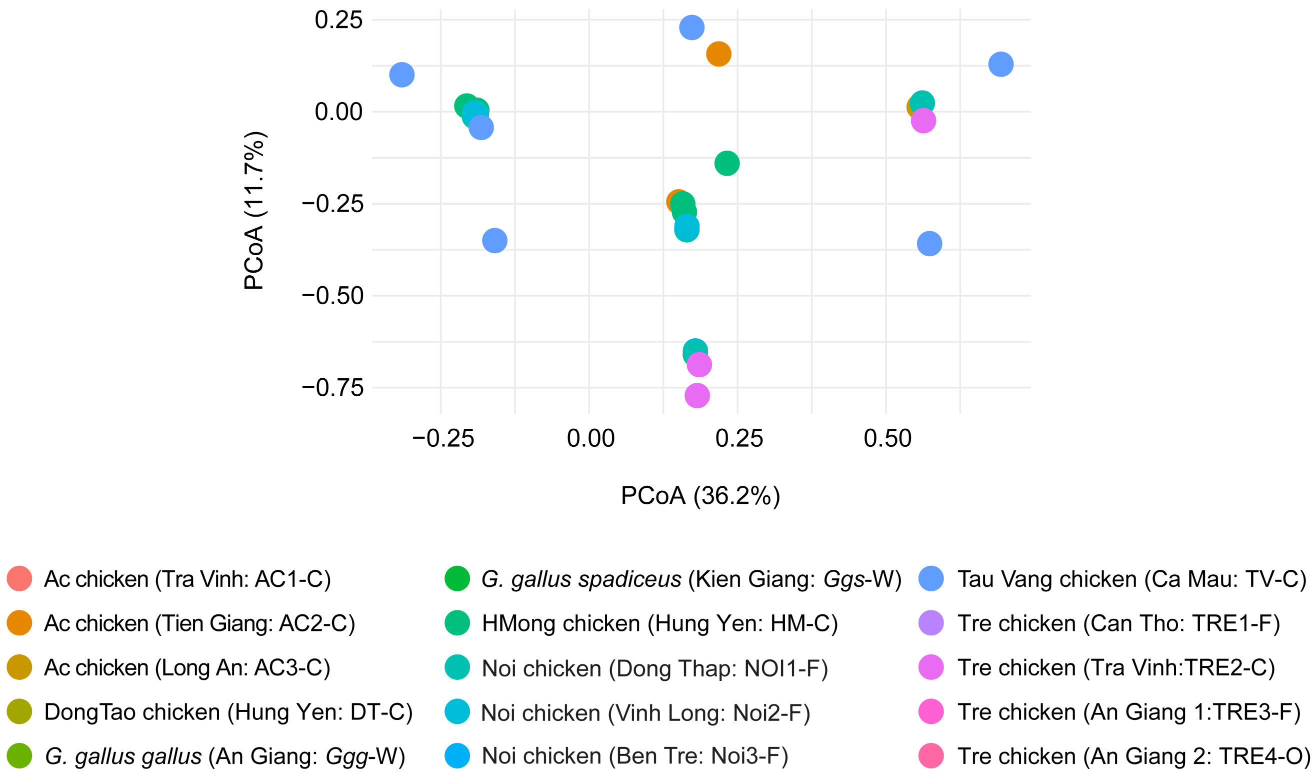

Supplementary Figure 2. Genetic differentiation among populations was evaluated using Principal Coordinate Analysis (PCoA) based on allele frequency data.

*Blec2*VN1*	C	P	E	T	N	G	R	N	G	T	E	L	C	E	N	T	S	I	E	Q	H	F	C	Q	S	T	W	N	S	S	A
*Blec2*VN2*	C	P	E	T	N	G	R	N	G	T	E	L	C	K	N	T	S	I	E	Q	Y	F	C	Q	S	K	L	S	S	S	A
*Blec2*VN3*	C	P	E	T	N	G	R	N	G	T	E	L	C	K	N	T	S	I	E	Q	Y	F	C	Q	S	K	W	S	S	S	A
*Blec2*VN4*	C	P	E	T	N	G	R	N	G	T	E	L	C	E	N	T	S	T	E	Q	H	F	C	Q	S	T	W	N	S	S	A
*Blec2*VN5*	C	P	E	T	N	G	R	N	G	T	E	L	C	E	N	T	S	I	E	Q	Y	F	C	Q	S	T	W	N	S	S	A
*Blec2*VN6*	C	P	E	T	N	G	R	N	G	T	E	L	C	E	N	T	S	T	E	Q	H	F	C	Q	S	T	W	N	S	S	A
*Blec2*VN7*	C	P	E	T	N	G	R	N	G	T	E	L	C	E	N	T	S	I	E	Q	Y	F	C	Q	S	K	W	S	S	S	A
*Blec2*VN8*	C	P	E	T	N	G	R	N	G	T	E	L	C	K	N	T	S	T	E	Q	Y	F	C	Q	S	K	W	S	S	S	A
*Blec2*VN9*	C	P	E	T	N	G	R	N	G	T	E	L	C	K	N	T	S	I	E	Q	H	F	C	Q	S	K	W	S	S	S	A
*Blec2*VN10*	C	P	E	T	N	G	R	N	-	T	E	L	C	K	N	T	S	I	E	Q	Y	F	C	Q	S	K	R	S	S	S	A
*Blec2*VN11*	C	P	E	T	N	G	R	N	G	T	E	L	C	K	N	T	S	I	E	Q	Y	F	C	Q	S	T	W	N	S	S	A
Breed Ghagus (ASY04180)	C	P	E	T	N	G	R	N	G	T	E	L	C	K	N	T	S	I	E	Q	H	F	C	Q	S	K	W	S	S	S	A
Breed White_Leghorn B15 (BAG69304)	C	P	E	T	N	G	R	N	G	T	E	L	C	K	N	T	S	I	E	Q	H	F	C	Q	S	K	W	S	S	S	A
Breed White_Leghorn B19 (CAO00523)	C	P	E	T	N	G	R	N	G	T	E	L	C	E	N	T	S	I	E	Q	Y	F	C	Q	S	T	W	N	S	S	A
Breed Korea chicken (WBF70131)	C	P	E	T	N	G	R	N	G	T	E	L	C	E	N	T	S	I	E	Q	Y	F	C	Q	S	T	W	N	S	S	A
Breed Cornell (BAG69429)	C	P	E	T	N	G	R	N	G	T	E	L	C	E	N	T	S	I	E	Q	Y	F	C	Q	S	T	W	N	S	S	A
Breed Ancona and White Leghorn cross (ACY01469)	C	P	E	T	N	G	R	N	G	T	E	L	C	K	N	T	S	I	E	Q	Y	F	C	Q	S	K	W	S	S	S	A

Supplementary Figure 3. Alignment of the amino acid sequences of the *Blec2* gene alleles from partial exon 4 and various chicken breed sequences retrieved from the NCBI database.

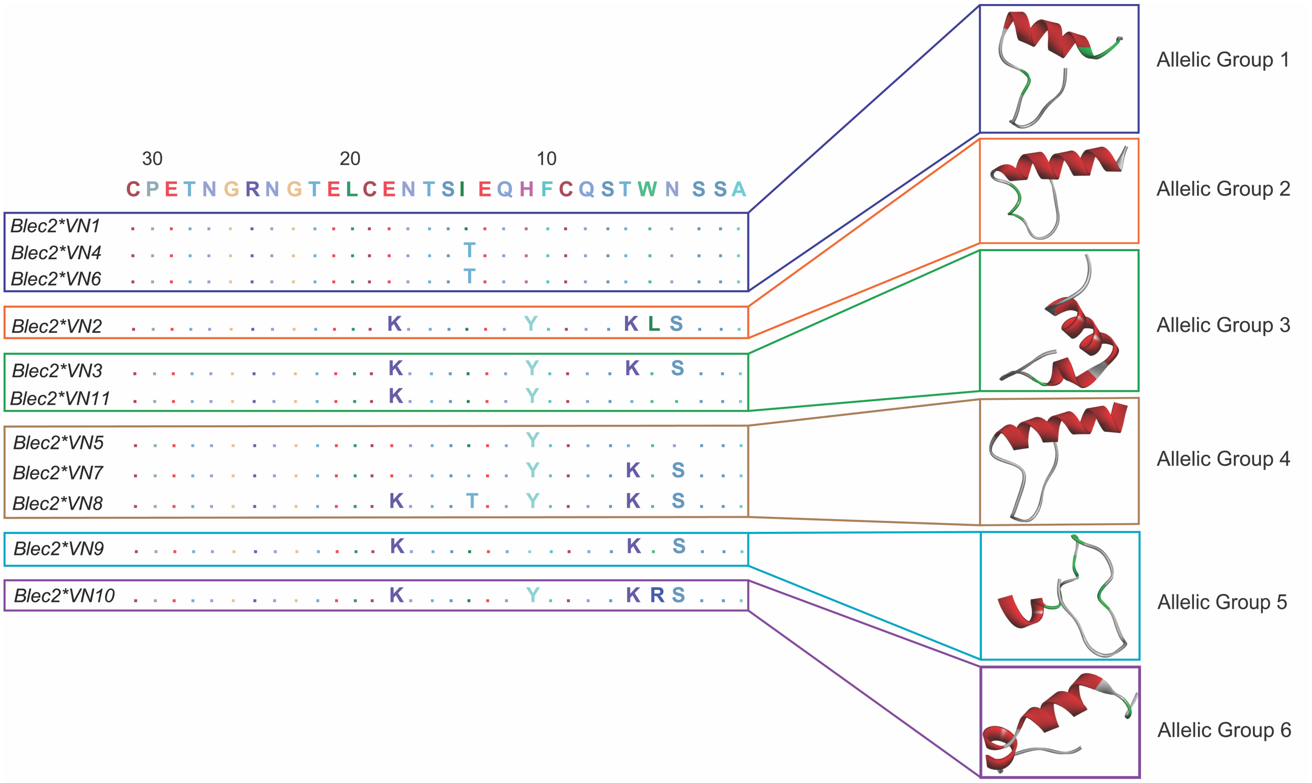

Supplementary Figure 4. The 3D structure of the publicly available amino acid sequences (A5HUL0) of partial *Blec2* protein encoded by exon 4 of the *Blec2* gene and alpha-fold protein structures of lectin-like natural killer cell surface protein domain in *Blec2* gene alleles. Three types of alpha-fold protein structures (highlighted in red) are superimposed on the reference protein structure.

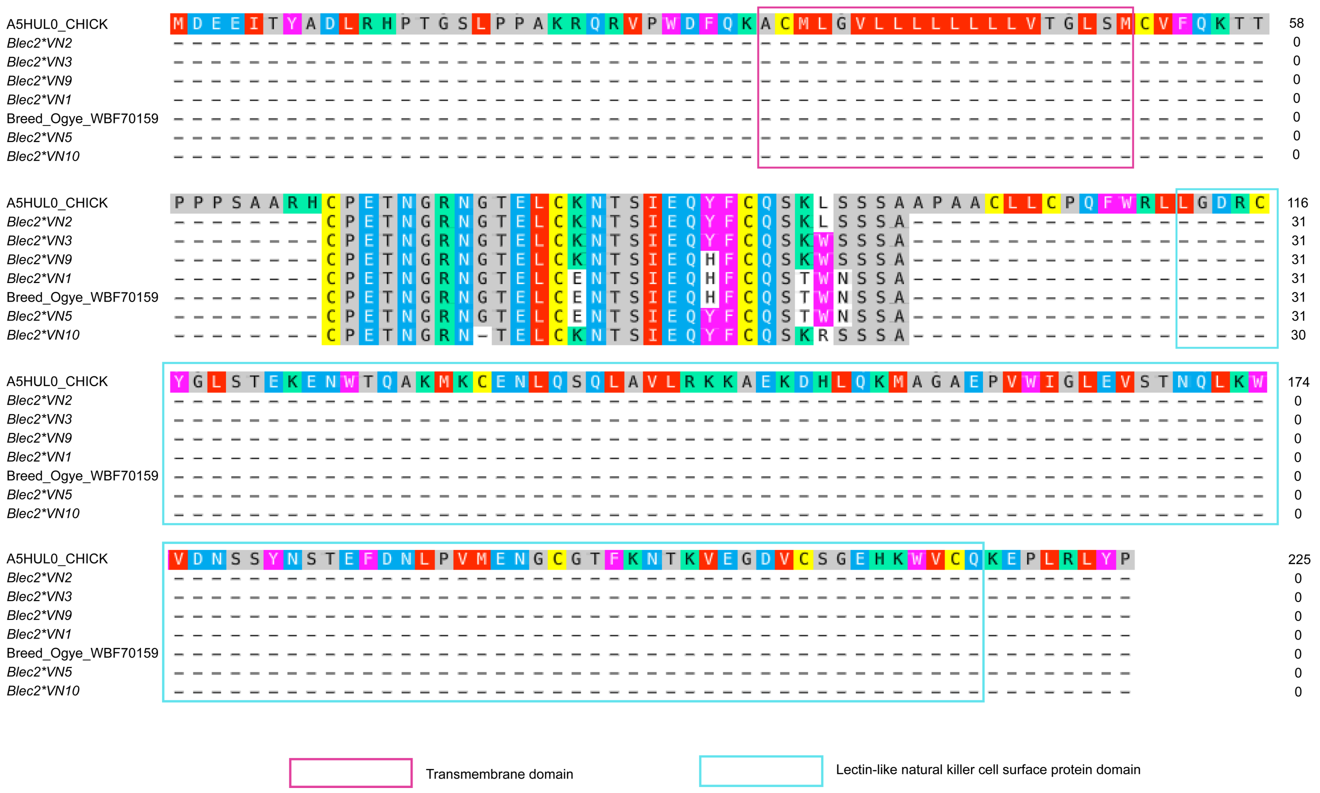

Supplementary Figure 5. Alignment of amino acid sequences of partial exon 4 of the *Blec2* gene alleles with the reference sequence (A5HUL0). The transmembrane domain and lectin-like natural killer cell surface protein domain are indicated using pink- and blue-colored squares, respectively.

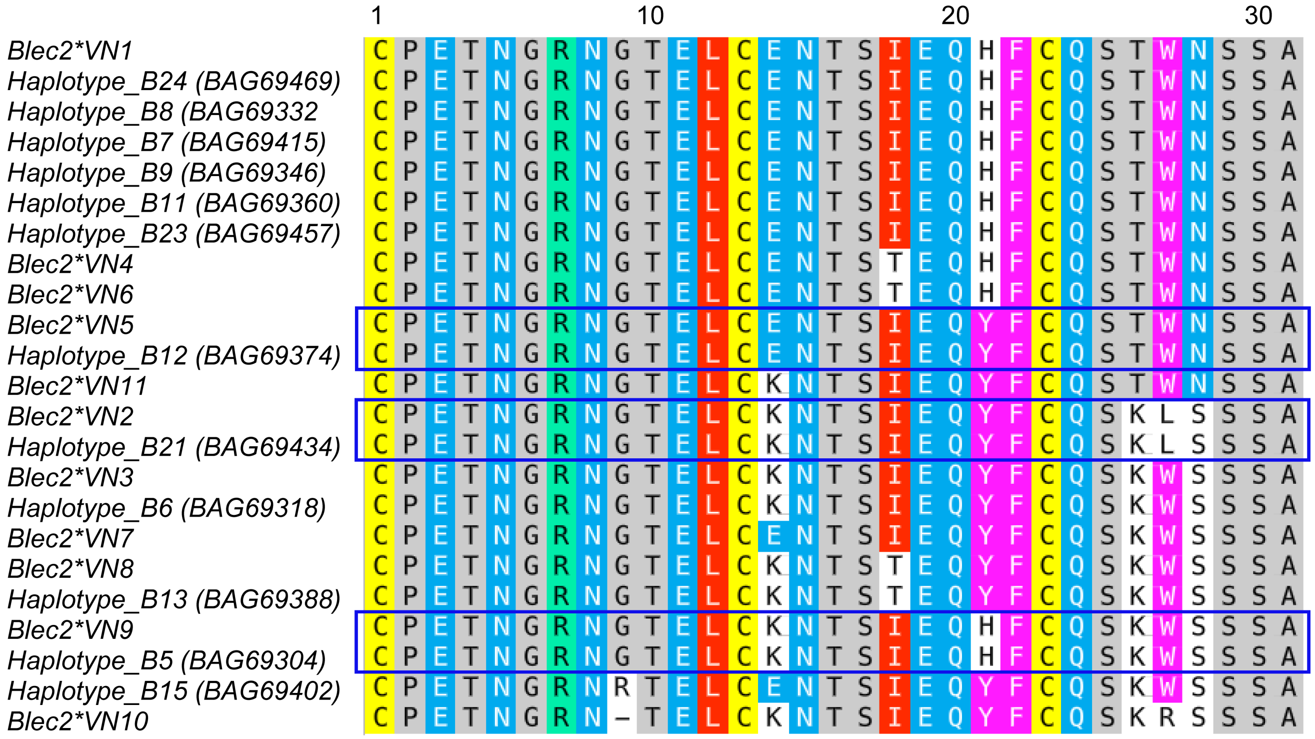

Supplementary Figure 6. Multiple sequence alignment of *Blec2* amino acid sequences. Newly identified variants (*Blec2*VN1-11*) are compared with known reference haplotypes. Blue boxes indicate complete sequence identity between the novel alleles and established references.

1.2 Supplementary Tables

Supplementary Table 1. Detailed information on the specimens from 12 populations of Noi, Tre, Hmong, Dong Tao, and Tau Vang chicken breeds and red junglefowl

Breeds	Population	Purpose	Abbreviation	Sample (*N*)
Ac	Tra Vinh	Consumption	AC-C	20
	Tien Giang	Consumption	AC-C	20
	Long An	Consumption	AC-C	20
Noi	Dong Thap	Fighting	NOI1-F	15
	Vinh Long	Fighting	NOI2-F	15
	Ben Tre	Fighting	NOI3-F	7
Tre	Can Tho	Fighting	TRE1-F	5
	Tra Vinh	Consumption	TRE2-C	13
	An Giang1	Fighting	TRE3-F	8
	An Giang2	Ornamental	TRE4-O	5
Hmong	Hung Yen	Consumption	HM-C	15
Dong Tao	Hung Yen	Consumption	DT-C	11
Tau Vang	Ca Mau	Conservation	TV-Con	15
G. gallus spadiceus	Kien Giang	Wild	*Ggs*-W	10
G. gallus gallus	An Giang	Wild	*Ggg*-W	3
Total				182

Supplementary Table 2. Distribution of *Blec2* gene alleles in Vietnamese indigenous and local chicken breeds and Red junglefowl

Breed/ red junglefowl subspecies	Population	Alleles										
		VN1	VN2	VN3	VN4	VN5	VN6	VN7	VN8	VN9	VN10	VN11
Ac	Tra Vinh	✓	✓	✓	✓	-	-	-	-	-	-	-
	Tien Giang	✓	✓	✓	✓	✓	✓	✓	✓	-	-	-
	Long An	✓	✓	✓	-	-	-	-	✓	-	-	-
Noi	Dong Thap	✓	✓	✓	✓	✓	-	-	✓	-	✓	-
	Vinh Long	✓	✓	✓	✓	-	-	✓	✓	-	-	-
	Ben Tre	✓	✓	✓	-	-	-	✓	-	-	-	-
Tre	Can Tho	✓	✓	✓	-	-	-	-	-	-	-	-
	Tra Vinh	✓	✓	✓	✓	-	-	-	-	-	-	-
	An Giang1	✓	✓	✓	-	-	-	-	-	-	-	-
	An Giang2	✓	-	✓	-	-	-	-	-	-	-	-
Hmong	Hung Yen	✓	✓	✓	-	✓	-	-	✓	✓	✓	✓

Breed/ red junglefowl subspecies	Population	Alleles										
		VN1	VN2	VN3	VN4	VN5	VN6	VN7	VN8	VN9	VN10	VN11
Dong Tao	Hung Yen	✓	✓	✓	-	-	-	-	✓	-	-	-
Tau Vang	Ca Mau	✓	✓	✓	✓	-	-	-	-	-	-	-
G. gallus spadiceus	Kien Giang	✓	-	✓	-	-	-	-	-	-	-	-
G. gallus gallus	An Giang	✓	✓	✓	-	-	-	-	-	-	-	-
Indigenous and local breeds		✓	✓	✓	✓	✓	✓	✓	✓	✓	✓	✓
Red junglefowl		✓	✓	✓	-	-	-	-	-	-	-	-

Supplementary Table 3. Mutation types and their locations in the partial fragments of the *Blec2* gene exon 4. Sequences from Vietnamese indigenous and local chicken breeds and Red junglefowl were compared with the reference sequence (accession number: OM953777)

No	Allele	Position of mutation and nucleotide substitution	Mutation type	Amino acid change (nucleotide substitution)
1	*Blec2*VN6*	9648T>C	Silent	-
2	*Blec2*VN2, Blec2*VN3, Blec2*VN7, Blec2*VN8, Blec2*VN9, Blec2*VN10, Blec2*VN11*	9671G>A	Silent	-
3	*Blec2*VN2, Blec2*VN3, Blec2*VN7, Blec2*VN8, Blec2*VN9, Blec2*VN10*	9695T>C	Missense	Asparagine to Serine (GTT to GCT)
4	*Blec2*VN2*	9698C>A	Missense	Tryptophan to Leucine, (CCA to CTA)
5	*Blec2*VN10*	9699A>T	Missense	Tryptophan to Arginine, (CCA to CCT)
6	*Blec2*VN2, Blec2*VN3, Blec2*VN7, Blec2*VN8, Blec2*VN9, Blec2*VN10*	9701G>T	Missense	Threonine to Lysine, (TGT to TTT)
**7**	*Blec2*VN2, Blec2*VN3, Blec2*VN5, Blec2*VN7, Blec2*VN8, Blec2*VN10, Blec2*VN11*	9717G>A	Missense	Histidine to Tyrosine, (GTG to GTA)
8	*Blec2*VN4, Blec2*VN6, Blec2*VN8*	9725A>G	Missense	Isoleucine to Threonine, (AAT to AGT)
9	*Blec2*VN2, Blec2*VN3, Blec2*VN8, Blec2*VN9, Blec2*VN10, Blec2*VN11*	9738C>T	Missense	Glutamic acid to Lysine (CTC to CTT)
10	*Blec2*VN10*	9753Delete C	Silent	-

Note: *The Blec2* gene is located on the negative strand; thus, the genomic mutation corresponds to the reverse complement of the intended coding change

Supplementary Table 4. Detailed site-by-site results from the MEME analysis based on the alleles of the *Blec2* gene

Part	Codon	α	β1	p1	β+	p+	LRT	*p*-value	Branches under selection	q	Class
1	1	0.000	0.000	1.000	0.000	0.000	0.000	1.000	0	1	Invariable
1	2	0.000	0.000	1.000	0.000	0.000	0.000	1.000	0	1	Invariable
1	3	0.000	0.000	1.000	0.000	0.000	0.000	1.000	0	1	Invariable
1	4	0.003	0.000	0.001	25.872	0.999	0.782	0.361	1	1	Neutral
1	5	44.856	35.143	0.809	29.367	0.191	0.000	0.667	0	1	Neutral
1	6	0.007	0.000	0.001	21.118	0.999	0.346	0.475	1	1	Neutral
1	7	0.000	0.000	1.000	0.000	0.000	0.000	1.000	0	1	Invariable
1	8	0.000	0.000	1.000	0.000	0.000	0.000	1.000	0	1	Invariable
1	9	0.000	0.000	1.000	0.000	0.000	0.000	1.000	0	1	Invariable
1	10	0.000	0.000	1.000	0.000	0.000	0.000	1.000	0	1	Invariable
1	11	78.05	0.000	0.994	1.290	0.006	0.000	0.667	0	1	Neutral
1	12	0.000	0.000	1.000	0.000	0.000	0.000	1.000	0	1	Invariable
1	13	0.000	0.000	1.000	0.000	0.000	0.000	1.000	0	1	Invariable
1	14	0.001	0.000	0.010	19.427	0.99	0.348	0.475	2	1	Neutral
1	15	0.000	0.000	1.000	0.000	0.000	0.000	1.000	0	1	Invariable
1	16	0.000	0.000	1.000	0.000	0.000	0.000	1.000	0	1	Invariable
1	17	0.000	0.000	1.000	0.000	0.000	0.000	1.000	0	1	Invariable

Part	Codon	α	β1	p1	β+	p+	LRT	*p*-value	Branches under selection	q	Class
1	18	115.872	0.000	1.000	3.73	0.000	0.000	0.667	0	1	Neutral
1	19	0.000	0.000	1.000	0.000	0.000	0.000	1.000	0	1	Invariable
1	20	0.000	0.000	1.000	0.000	0.000	0.000	1.000	0	1	Invariable
1	21	0.000	0.000	1.000	0.000	0.000	0.000	1.000	0	1	Invariable
1	22	0.000	0.000	1.000	0.000	0.000	0.000	1.000	0	1	Invariable
1	23	0.000	0.000	1.000	0.000	0.000	0.000	1.000	0	1	Invariable
1	24	0.000	0.000	1.000	0.000	0.000	0.000	1.000	0	1	Invariable
1	25	0.000	0.000	1.000	0.000	0.000	0.000	1.000	0	1	Invariable
1	26	0.000	0.000	1.000	0.000	0.000	0.000	1.000	0	1	Invariable
1	27	0.000	0.000	1.000	0.000	0.000	0.000	1.000	0	1	Invariable
1	28	0.000	0.000	1.000	0.000	0.000	0.000	1.000	0	1	Invariable
1	29	0.000	0.000	1.000	0.000	0.000	0.000	1.000	0	1	Invariable
1	30	0.000	0.000	1.000	0.000	0.000	0.000	1.000	0	1	Invariable
1	31	0.000	0.000	1.000	0.000	0.000	0.000	1.000	0	1	Invariable

α: Synonymous substitution rate (silent mutations); β1: Non-synonymous substitution rate for the first site class (typically purifying selection); p1: Proportion of the site belonging to class β1; β^+: Non-synonymous substitution rate for the positive/diversifying selection class; p^+: proportion of the site belonging to the positive selection class β^+; LRT: Likelihood Ratio Test statistic; *p*-value: The statistical probability of the LRT result occurring by chance. Values < 0.05 indicate significance; q-value: The *p*-value corrected for multiple testing (False Discovery Rate). Reliable results require $q < 0.05$; Class: The evolutionary category assigned to the codon.

Supplementary Table 5. Detailed site-by-site results from the FEL analysis based on the alleles of the *Blec2* gene

Partition	codon	α	β	α=β	LRT	*p*-value	Total branch length	class
1	1	0.000	0.000	0.000	0.000	1.000	0.000	Invariable
1	2	0.000	0.000	0.000	0.000	1.000	0.000	Invariable
1	3	0.000	0.000	0.000	0.000	1.000	0.000	Invariable
1	4	0.000	3.880	2.567	0.784	0.376	0.418	Neutral
1	5	6.350	4.827	5.462	0.035	0.851	0.890	Neutral
1	6	0.000	2.891	2.426	0.348	0.555	0.396	Neutral
1	7	0.000	0.000	0.000	0.000	1.000	0.000	Invariable
1	8	0.000	0.000	0.000	0.000	1.000	0.000	Invariable
1	9	0.000	0.000	0.000	0.000	1.000	0.000	Invariable
1	10	0.000	0.000	0.000	0.000	1.000	0.000	Invariable
1	11	11.297	0.000	4.370	3.616	0.057	0.712	Neutral
1	12	0.000	0.000	0.000	0.000	1.000	0.000	Invariable
1	13	0.000	0.000	0.000	0.000	1.000	0.000	Invariable
1	14	0.000	2.773	2.542	0.350	0.554	0.414	Neutral
1	15	0.000	0.000	0.000	0.000	1.000	0.000	Invariable
1	16	0.000	0.000	0.000	0.000	1.000	0.000	Invariable
1	17	0.000	0.000	0.000	0.000	1.000	0.000	Invariable
1	18	16.668	0.000	5.845	3.980	0.046	0.953	Neutral

Partition	codon	α	β	α=β	LRT	*p*-value	Total branch length	class
1	19	0.000	0.000	0.000	0.000	1.000	0.000	Invariable
1	20	0.000	0.000	0.000	0.000	1.000	0.000	Invariable
1	21	0.000	0.000	0.000	0.000	1.000	0.000	Invariable
1	22	0.000	0.000	0.000	0.000	1.000	0.000	Invariable
1	23	0.000	0.000	0.000	0.000	1.000	0.000	Invariable
1	24	0.000	0.000	0.000	0.000	1.000	0.000	Invariable
1	25	0.000	0.000	0.000	0.000	1.000	0.000	Invariable
1	26	0.000	0.000	0.000	0.000	1.000	0.000	Invariable
1	27	0.000	0.000	0.000	0.000	1.000	0.000	Invariable
1	28	0.000	0.000	0.000	0.000	1.000	0.000	Invariable
1	29	0.000	0.000	0.000	0.000	1.000	0.000	Invariable
1	30	0.000	0.000	0.000	0.000	1.000	0.000	Invariable
1	31	0.000	0.000	0.000	0.000	1.000	0.000	Invariable

α: Synonymous substitution rate (silent mutations); β1: Non-synonymous substitution rate (amino acid changing mutations); α = β: the estimated substitution rate under the null hypothesis of neutral evolution; LRT: likelihood ratio test; *p*-value: The statistical probability of the LRT result occurring by chance. Values < 0.05 indicate significance; Class: The evolutionary category assigned to the codon.

Supplementary Table 6. Detailed site-by-site results from the FUBAR analysis based on the alleles of the *Blec2* gene

Site	Partition	α	β	β−α	Prob [α>β]	Prob [α<β]	BayesFactor [α<β]
1	1	1.992	0.703	-1.289	0.482	0.463	0.954
2	1	1.864	1.379	-0.485	0.408	0.528	1.238
3	1	12.276	1.109	-11.167	0.819	0.143	0.185
4	1	9.186	0.938	-8.248	0.825	0.139	0.178
5	1	2.390	2.253	-0.137	0.303	0.646	2.022
6	1	6.424	1.710	-4.714	0.476	0.478	1.013
7	1	2.175	1.359	-0.816	0.348	0.600	1.656
8	1	1.866	0.951	-0.915	0.513	0.427	0.823
9	1	0.741	0.541	-0.200	0.574	0.356	0.612
10	1	1.987	0.962	-1.025	0.460	0.476	1.005
11	1	1.497	15.536	14.039	0.033	0.941	17.574
12	1	1.873	0.904	-0.969	0.562	0.378	0.672
13	1	18.764	0.855	-17.909	0.968	0.02	0.023
14	1	4.717	1.295	-3.421	0.475	0.474	0.995
15	1	1.102	0.865	-0.237	0.401	0.535	1.272
16	1	1.861	0.742	-1.118	0.509	0.427	0.825
17	1	1.635	1.035	-0.600	0.508	0.437	0.859

Site	Partition	α	β	β−α	Prob [α>β]	Prob [α<β]	BayesFactor [α<β]
18	1	1.455	15.732	14.277	0.045	0.928	14.155
19	1	0.699	0.786	0.087	0.367	0.578	1.516
20	1	1.135	0.773	-0.362	0.383	0.561	1.415
21	1	1.133	1.228	0.095	0.516	0.423	0.810
22	1	1.239	1.275	0.036	0.421	0.514	1.170
23	1	1.78	0.726	-1.054	0.488	0.447	0.895
24	1	0.876	0.47	-0.406	0.537	0.377	0.670
25	1	1.208	1.415	0.207	0.375	0.560	1.408
26	1	1.537	0.795	-0.742	0.475	0.472	0.988
27	1	5.346	2.017	-3.329	0.407	0.544	1.319
28	1	1.563	1.671	0.108	0.372	0.573	1.486
29	1	3.459	0.873	-2.586	0.561	0.383	0.686
30	1	1.237	0.547	-0.69	0.616	0.32	0.520
31	1	1.809	0.75	-1.059	0.516	0.426	0.821

α: Synonymous substitution rate (silent mutations); β1: Non-synonymous substitution rate for the first site class (amino acid changing mutations); β – α: The difference between the rates. Positive values indicate β > α (potential positive selection); negative values indicate α > β (purifying selection); Prob [α > β]: The posterior probability that the site is under negative (purifying) selection. Values > 0.90 are generally considered significant; Prob [α < β]: The posterior probability that the site is under positive (diversifying) selection; BayesFactor [α < β]: The Bayes Factor indicating the strength of evidence for positive selection.
